# Triglyceride-glucose index predicts death in patients with stroke younger than 65

**DOI:** 10.3389/fneur.2023.1198487

**Published:** 2023-08-03

**Authors:** Ruishan Liu, Lijuan Li, Lu Wang, Shihong Zhang

**Affiliations:** ^1^Center of Cerebrovascular Diseases, Department of Neurology, West China Hospital, Sichuan University, Chengdu, China; ^2^Rehabilitation Medicine Center and Institute of Rehabilitation Medicine, West China Hospital, Sichuan University, Chengdu, China; ^3^Key Laboratory of Rehabilitation Medicine in Sichuan Province, Chengdu, China

**Keywords:** triglyceride-glucose index, insulin resistance, acute ischemic stroke, age, prognosis, death

## Abstract

**Background:**

The triglyceride-glucose index (TGI), a reliable surrogate indicator of insulin resistance (IR), has been proven to be a predictor of the incidence of ischemic stroke. The role of TGI in predicting the outcomes of stroke patients remains controversial. Susceptibility to IR-related diseases varies among patients of different ages. The study aims to evaluate the predictive value of TGI levels on clinical outcomes of patients with ischemic stroke of different ages.

**Method:**

This was a retrospective cohort study including patients with ischemic stroke in the Department of Neurology at West China Hospital. TGI was calculated as ln [fasting triglyceride (mg/dL) × fasting glucose (mg/dL)/2]. The patients were subdivided into 3 tertiles according to TGI levels. Multivariate logistic regression analyses were conducted to estimate the association between TGI levels and post-stroke outcomes among the whole patients, younger patients (<65), and older patients (>=65). The outcomes included death and unfavorable functional outcome (modified Rankin scale score 3–6) at 3 and 12 months after stroke.

**Results:**

A total of 3,704 patients (men, 65.08%, mean age, 61.44 ± 14.15; women 34.92%, mean age, 65.70 ± 13.69) were enrolled in this study. TGI levels were not associated with 3 month or 12 month death in the whole patients. Patients with higher TGI levels (T2 and T3) had a higher risk of 3 month death than those had lower TGI levels (T1) in the younger group (T2 vs. T1: OR 2.64, 95% CI 1.03–6.79, *p* = 0.043; T3 vs. T1: OR 2.69, 95% CI 1.00–7.10, *p* = 0.049) but not in the older group. Additionally, Kaplan–Meier estimate analysis illustrated that the 12 month death risk was significantly higher in the group with the highest TGI among younger patients (*p* for log-rank test = 0.028) but not among older patients. There was an interactive effect between TGI and age on 3 month death (*p* for interaction = 0.013) and 12 month death (*p* for interaction = 0.027). However, TGI was not associated with unfavorable functional outcome at 3 month or 12 month after stroke.

**Conclusion:**

Elevated TGI independently predicts death at 3 months and 12 months in patients under 65 with ischemic stroke. Regulating TGI is expected to be an approach to enhance prognosis in young individuals affected by ischemic stroke.

## 1. Introduction

Stroke is a significant cause of death and long-term disability representing approximately 10% of global deaths (1). Ischemic stroke comprises more than 80% of all stroke cases and becomes a growing burden on the global healthcare system (2). Previous studies have identified several risk factors playing a crucial role in predicting patient prognosis, such as age, sex, atrial fibrillation, hypertension, and congenital heart disease (3). Moreover, certain blood biomarkers serve as valuable prognostic indicators for post-stroke death risk. These biomarkers include inflammatory factors (e.g., tumor necrosis factor-α, soluble tumor necrosis factor receptor) and blood coagulation biomarkers (e.g., von Willebrand factor, platelet count) (4). Adequate risk stratification facilitates the implementation of effective secondary preventive measures and enhances the quality of life for individuals with stroke (3). Nevertheless, modifying many of these factors is challenging, resulting in limited effects on improving outcomes. Therefore, a crucial requirement exists for identifying modifiable predictors that can help identify patients with poor outcomes and optimize the management of individuals with ischemic stroke.

Insulin resistance (IR), which is characterized by reduced insulin sensitivity, could lead to an insufficient physiological response to a normal amount of insulin. Strong evidence has demonstrated the pivotal role of IR in the pathogenesis of stroke, particularly through embolism and atherosclerosis (5–9). The hyperinsulinemic-euglycemic clamp is the gold standard test for measuring IR; however, its widespread application in clinical settings is challenging due to its high cost and complex detection process (10). Recent accumulating evidence suggests that the triglyceride glucose index (TGI), derived from triglyceride and glucose levels, is a promising surrogate marker for IR (11–13). Multiple studies have confirmed the strong association between TGI and the hyperinsulinemic-euglycemic clamp (9), and it has even demonstrated superior performance to the homeostasis model assessment of insulin resistance (HOMA-IR) for evaluating IR (12, 14).

Recent epidemiological research has provided substantial evidence that supported the relationship between IR and an elevated risk of stroke (15–18). Several prior studies found that it might serve as a predictor for the occurrence of acute ischemic stroke (19–22). However, there is a scarcity of studies investigating the role of TGI in predicting prognosis among individuals following ischemic stroke, and the results obtained thus far have been inconsistent (23–26).

According to a previous study, younger individuals with elevated TGI levels may be more vulnerable to diseases and death associated with IR (27). We hypothesized that age might influence the predictive effect of TGI on the clinical outcome after a stroke, serving as a significant interactive factor contributing to the controversy observed in previous studies. However, no evidence was found to support the influence of age on the association between TGI and the prognosis of ischemic stroke. Consequently, this comprehensive retrospective cohort study aimed to examine the predictive value of TGI levels in determining the clinical outcomes of patients with ischemic stroke. Moreover, it sought to explore the underlying influence of age on the association between TGI and stroke prognosis, marking the first investigation of its kind.

## 2. Method

### 2.1. Study population

This study is based on the Chengdu Stroke Registry that included patients with stroke at West China Hospital from 1 January 2012 to 31 December 2018. The inclusion criteria included (1) diagnosed with acute ischemic stroke according to the 1989 criteria of the WHO based on clinical characteristics and imaging manifestations (28); (2) ≥18 years old; and (3) time from onset to admission within 7 days. The exclusion criteria included (1) loss of follow-up information; and (2) inconclusive diagnosis of ischemic stroke.

### 2.2. Data collection

We collected patient information in terms of demographic data, vascular risk factors, baseline National Institute of Health Stroke Scale (NIHSS), subgroup of stroke according to Trial of ORG 10172 in Acute Stroke Treatment (TOAST) (29), in-hospital medications and therapy after admission, laboratory tests and follow-up information. The vascular risk factors consisted of a history of hypertension, diabetes, atrial fibrillation, rheumatic heart disease, coronary heart disease, stroke, a history of regular smoking or drinking (30–33). Severe stroke was defined as an NIHSS score higher than 15 (34).

Blood sampling was conducted within 24 h after admission to make the data representative and comparable. TGI was calculated as ln [fasting triglyceride (mg/dL) × fasting glucose (mg/dL)/2] (35).

Follow-up information was obtained from telephone interviews conducted by trained physicians at 3 and 12 months after admission.

### 2.3. Outcome measures

The primary outcome was 3 month death from any cause. The secondary outcomes included 3 month unfavorable functional outcomes, 12 month death, and 12 month unfavorable functional outcomes. Unfavorable functional outcomes were defined as an mRS score of 3 to 6, including moderate to severe disability that require partial or full assistance with daily living, or death (36).

### 2.4. Statistical analysis

The baselines were presented as the younger group and the older group according to the cutoff age of 65 years old. Continuous variables are described as the means with standard deviations (SDs) or medians with the interquartile range (IQR). Categorical variables are described as numbers and percentages.

Multivariate logistic regression models were utilized to calculate odds ratios (ORs) and their 95% confidence intervals (CIs) of TGI as a categorical variable, using the first TGI tertile as the reference predicting different outcomes in all patients, the younger patients, and the older patients. The variables included in multivariate logistic regression were those with statistical differences in univariate analysis and important factors that had an impact on outcomes in clinical practice. To examine the interactive effect of TGI and age on outcomes, *P* for trend was calculated.

Then, we used Kaplan–Meier curves to predict the association between TGI and 12 month death. Log-rank test was conducted to test differences in death rates. Finally, a cubic spline curve was described to show the dose–response correlation between TGI and death in all patients, young patients, and old patients, with the 50th percentile of the TGI distribution serving as the reference (37).

To examine the interactive effect of other factors on the association between TGI and 3 month death, subgroup analysis was also performed in mild and severe stroke, male and female patients, patients with and without atrial fibrillation, rheumatic heart disease, coronary heart disease, diabetes, hypertension, cardioembolic stroke subtype, lipid lowering therapy, antidiabetic drugs, and cancer. *P* for interaction was derived from the likelihood-ratio test. All the data were analyzed using Stata version 15.0 software (STATA Corporation, College Station, TX). A two-sided *p* value <0.05 was considered statistically significant.

## 3. Results

### 3.1. Baseline characteristics

After excluding 268 patients without laboratory values and 480 patients without information on follow-up, 3,704 participants were available for the final analysis. The baseline characteristics of the young and old patients are shown in Tables 1, 2, respectively.

**Table 1:** Baseline characteristics of included patients grouped by TGI level in patients younger than 65 years old.

Variables	T1 (5.91–8.50)	T2 (8.50–9.04)	T3 (9.04–12.90)	*p*-value
*N*	549	617	677	–
Age, years, mean (SD)	49.36 ± 11.16	51.99 ± 9.36	52.78 ± 8.19	**<0.001**
Male, *n* (%)	362 (65.94)	423 (68.56)	467 (68.98)	0.470
Baseline NIHSS score, median (Q1–Q3)	4 (1–8)	4 (2–9)	4 (2–8)	0.270
Time from onset to admission, hour, median (Q1–Q3)	48 (24–120)	72 (24–120)	72 (24–120)	0.974
Hypertension, *n* (%)	205 (37.34)	316 (51.22)	431 (63.66)	**<0.001**
Diabetes, *n* (%)	31 (5.65)	91 (14.75)	299 (44.17)	**<0.001**
Atrial fibrillation, *n* (%)	60 (10.93)	55 (8.91)	31 (4.58)	**<0.001**
Rheumatic heart disease, *n* (%)	59 (10.75)	51 (8.27)	27 (3.99)	**<0.001**
Coronary heart disease, *n* (%)	18 (3.28)	12 (1.94)	23 (3.40)	0.213
History of stroke, *n* (%)	52 (9.47)	58 (9.40)	67 (9.90)	0.887
Drinking, *n* (%)	156 (28.42)	218 (35.33)	241 (35.60)	**0.010**
Smoking, *n* (%)	217 (39.53)	307 (49.76)	308 (45.50)	**0.003**
TOAST				**<0.001**
Large-artery atherosclerosis, *n* (%)	180 (32.79)	248 (40.19)	251 (37.08)	
Small-artery occlusion, *n* (%)	92 (16.28)	100 (16.21)	172 (25.41)	
Cardio-embolism, *n* (%)	88 (15.58)	89 (14.42)	46 (6.79)	
Undetermined etiology, *n* (%)	33 (5.84)	23 (3.73)	25 (3.69)	
Other etiology, *n* (%)	172 (30.44)	157 (25.45)	183 (27.03)	
**Laboratory tests**				
Hemoglobin, g/L, mean (SD)	136.95 ± 21.31	140.13 ± 19.98	142.84 ± 19.88	0.143
platelet count, ^10^9^/L, mean (SD)	178.18 ± 84.69	180.48 ± 69.38	177.95 ± 67.73	**<0.001**
Creatinine, mg/dL, mean ± SD	75.79 ± 41.86	79.22 ± 35.24	84.52 ± 64.49	**<0.001**
Uric acid, μmol/L, mean ± SD	297.82 ± 93.66	312.86 ± 101.70	326.08 ± 109.74	**<0.001**
HDL, mmol/L, mean ± SD	1.32 ± 0.39	1.20 ± 0.40	1.12 ± 0.40	0.900
LDL, mmol/L, mean ± SD	2.14 (1.62–2.7)	2.49 (1.92–3.1)	2.79 (2.15–3.42)	**<0.001**
Total cholesterol, mmol/L, mean ± SD	3.81 (3.22–4.44)	4.22 (3.59–4.89)	4.76 (4.06–5.47)	**<0.001**
Glucose, mmol/L, mean ± SD	5.32 ± 1.15	6 ± 1.36	8.58 ± 4.46	**0.001**
Triglyceride, mmol/L, median (Q1–Q3)	0.89 (0.73–1.03)	1.38 (1.17–1.59)	2.21 (1.78–2.79)	**<0.001**
TGI, (mg/dL)^2^, mean ± SD	8.17 ± 0.29	8.76 ± 0.15	9.58 ± 0.53	**<0.001**
**Therapy after admission**				
Antiplatelet, *n* (%)	535 (94.69)	584 (94.65)	650 (96.01)	0.343
Thrombolysis, *n* (%)	11 (1.95)	14 (2.27)	15 (2.22)	0.940
Anticoagulation, *n* (%)	48 (8.50)	35 (5.67)	21 (3.10)	**<0.001**

SBP, systaltic blood pressure; DBP, diastolic blood pressure; SD, standard deviation. *The range of MHR and the number of patients in each tertile.

**Table 2:** Baseline characteristics of included patients grouped by low and high TGI group in patients older than 65 years old.

Variables	T1 (4.98–8.50)	T2 (8.50–9.04)	T3 (9.04–12.91)	*p*-value
*N*	682	614	565	–
Age, years, mean (SD)	75.02 ± 6.79	74.26 ± 6.21	74.06 ± 6.21	**0.020**
Male, *n* (%)	504 (73.90)	364 (59.28)	314 (55.58)	**<0.001**
Baseline NIHSS score, median (Q1–Q3)	5 (2–11)	5 (2–11)	5 (2–11)	0.800
Time from onset to admission, hour, median (Q1–Q3)	48 (24–96)	48 (20–108)	48 (20–96)	0.682
Hypertension, *n* (%)	502 (73.61)	481 (78.34)	493(87.26)	**<0.001**
Diabetes, *n* (%)	92 (13.49)	162 (26.38)	321 (56.81)	**<0.001**
Atrial fibrillation, *n* (%)	149 (21.85)	144 (23.45)	120 (21.24)	0.598
Rheumatic heart disease, *n* (%)	41 (6.01)	41 (6.68)	31 (5.49)	0.701
Coronary heart disease, *n* (%)	64 (9.38)	76 (12.38)	73 (12.92)	0.116
History of stroke, *n* (%)	145 (21.26)	134 (21.82)	101 (17.88)	0.166
Drinking, *n* (%)	151 (22.14)	115 (18.73)	95 (16.81)	0.051
Smoking, *n* (%)	197 (28.89)	154 (25.08)	138 (24.42)	0.144
TOAST				0.763
Large-artery atherosclerosis, *n* (%)	191 (28.01)	195 (31.76)	163 (28.85)	
Small-artery occlusion, *n* (%)	129 (18.91)	108 (17.59)	119 (21.06)	
Cardio-embolism, *n* (%)	146 (21.41)	133 (21.66)	112 (19.82)	
Undetermined etiology, *n* (%)	10 (1.47)	7 (1.14)	8 (1.16)	
Other etiology, *n* (%)	206 (30.21)	171 (27.85)	163 (28.85)	
**Laboratory tests**				
Hemoglobin, g/L, mean (SD)	130.16 ± 19.44	131.54 ± 18.26	131.30 ± 15.57	0.215
Platelet count, ^10^9^/L, mean (SD)	160.63 ± 76.65	166.24 ± 68.87	163.29 ± 63.35	**<0.001**
Creatinine, mg/dL, mean ± SD	85.75 ± 43.25	86.87 ± 44.73	91.50 ± 50.02	**<0.001**
Uric acid, μmol/L, mean ± SD	296.98 ± 102.07	309.89 ± 105.75	318.46 ± 110.00	0.175
HDL, mmol/L, mean ± SD	1.43 ± 0.42	1.32 ± 0.42	1.17 ± 0.36	**<0.001**
LDL, mmol/L, mean ± SD	2.08 (1.58–2.60)	2.45 (1.91–3.01)	2.62 (1.98–3.21)	**<0.001**
Total cholesterol, mmol/L, mean ± SD	3.82 (3.28–4.48)	4.25 (3.62–4.93)	4.52 (3.68–5.20)	**<0.001**
Glucose, mmol/L, mean ± SD	5.48 ± 1.35	6.58 ± 1.73	99.84 ± 5.71	**<0.001**
Triglyceride, mmol/L, median (Q1–Q3)	0.85 (0.69–1.00)	1.26 (1.04–1.49)	1.89 (1.48–2.49)	**<0.001**
TGI, (mg/dL)^2^, mean ± SD	8.15 ± 0.33	8.76 ± 0.15	9.56 ± 0.49	**<0.001**
**Therapy after admission**				
Antiplatelet, *n* (%)	640 (93.84)	581 (94.62)	535 (94.69)	0.761
Thrombolysis, *n* (%)	22 (3.23)	17 (2.77)	17 (3.01)	0.893
Anticoagulation, *n* (%)	36 (5.28)	34 (5.54)	29 (5.13)	0.951

SBP, systaltic blood pressure; DBP, diastolic blood pressure; SD, standard deviation. *The range of MHR and the number of patients in each tertile.

Among them, 1843 patients were younger than 65 years, with a median (IQR) TGI of 8.79 (8.42, 9.26). The TGI tertiles were divided into T1 (5.91–8.50), T2 (8.50–9.04), and T3 (9.04–12.90). Compared with T1 (49.36), the mean age in the T2 (51.99) and T3 groups (52.78) was older (*p* < 0.001). Additionally, 1861 patients were older than 65 years, with a median (IQR) TGI of 8.72 (8.34, 9.14). The mean ages in the T2 (74.26) and T3 (74.06) groups were slightly lower than that in the T1 group (75.02, *p* = 0.020).

In the younger group, patients with higher TGI tended to have more history of drinking (*p* = 0.010) and more comorbidities, including hypertension, diabetes, atrial fibrillation, and rheumatic heart disease (all *p* < 0.001). Based on TOAST classification, patients with higher TGI had a higher proportion of large-artery atherosclerosis but a lower proportion of cardioembolism subtype (*p* < 0.001). For laboratory serum tests, patients with higher TGI tended to have higher levels of creatinine, uric acid, LDL, total cholesterol, glucose, and triglycerides (all *p* = <0.001). Patients with higher TGI use less anticoagulation after admission (*p* < 0.001).

In the older group, patients with elevated TGI tended to have a lower proportion of males (*p* < 0.001) and more comorbidities, including hypertension and diabetes (both *p* < 0.001). Patients with different TGI levels have similar constituent structures of TOAST classification and similar therapy. The characteristics of laboratory tests were similar to those of the younger group, except that patients with higher TGI tended to have lower HDL levels (*p* < 0.001) but did not have higher hemoglobin or uric acid levels.

### 3.2. The predictive role of TGI on different outcomes in whole patients and different ages

#### 3.2.1. TGI and 3 month outcomes

To explore the effect of TGI in predicting outcomes in patients with different ages, logistic regression analysis was conducted for TGI tertiles using T1 as the reference in all, young, and old patients. Interactive analysis was utilised to explore the interactive effect between TGI and age (Table 3).

**Table 3:** Associations of TGI with outcomes in patients with different ages.

Outcomes	Groups	All patients		Younger patients		Older patients		*p* for interaction
		*n* (%)	OR (95%CI), *p*	*n* (%)	OR (95%CI), *p*	*n* (%)	OR (95%CI), *p*	
3 m death	T1	59(4.79)	Reference	7 (1.28)	Reference	52 (7.62)	Reference	**0.013**
	T2	58(4.71)	1.18 (0.78, 1.77), 0.435	20 (3.24)	2.64 (1.03, 6.79),**0.043**	38 (6.19)	0.95 (0.59, 1.53), 0.830	
	T3	56(4.51)	1.58 (1.04, 2.41), 0.032	23 (3.97)	2.69 (1.00, 7.10),**0.049**	33 (5.84)	1.43 (0.88, 2.32), 0.153	
	*P for trend*		0.032		0.068		0.165	
3 m unfavorable functional outcome	T1	381(30.95)	Reference	116 (21.13)	Reference	265 (38.86)	Reference	0.490
	T2	403(32.74)	1.06 (0.86, 1.31), 0.568	155 (25.12)	1.16 (0.84, 1.62), 0.371	248 (40.39)	1.06 (0.81, 1.41), 0.650	
	T3	399(32.13)	1.05 (0.84, 1.31), 0.653	173 (25.55)	1.23 (0.87, 1.73), 0.240	226 (40)	0.99 (0.73, 1.33), 0.946	
	*P for trend*		0.649		0.247		0.973	
12 m death	T1	96(7.80)	Reference	15 (2.73)	Reference	81 (11.88)	Reference	**0.027**
	T2	110(8.94)	1.28 (0.92, 1.79), 0.144	31 (5.02)	2.00 (0.99, 4.04), 0.052	79 (12.87)	1.19 (0.81, 1.76), 0.393	
	T3	104(8.37)	1.36 (0.95, 1.96), 0.094	36 (5.32)	2.52 (1.21, 5.22),**0.013**	68 (12.04)	1.15 (0.74, 1.77), 0.539	
	*P for trend*		0.091		**0.015**		0.509	
12 m unfavorable functional outcome	T1	262(21.28)	Reference	63 (11.48)	Reference	199 (29.18)	Reference	0.889
	T2	277(22.50)	1.17 (0.91, 1.49), 0.214	94 (15.24)	1.51 (1.00, 2.29), 0.049	183 (29.80)	1.11 (0.81, 1.51), 0.520	
	T3	259(20.85)	1.10 (0.85, 1.44), 0.470	90 (13.29)	1.33 (0.86, 2.07), 0.200	169 (29.91)	1.07 (0.76, 1.50), 0.714	
	*P for trend*		0.455		0.230		0.689	

Logistic regression analyses with adjustment for age, sex, baseline NIHSS, the time from stroke onset to admission, atrial fibrillation, rheumatic heart disease, coronary heart disease, drinking, smoking, TOAST, hemoglobin, creatinine, HDL, antiplatelet therapy, lipid lowering therapy and antidiabetic drugs, thrombolysis, and endovascular treatment.

Table 3 shows that in the whole patients, TGI levels were not associated with 3 month death. While in the younger group, elevated TGI (T2, T3) was associated with a higher risk of 3 month death (T2 vs. T1: OR 2.64, 95% CI 1.03–6.79, *p* = 0.043; T3 vs. T1: OR 2.69, 95% CI 1.00–7.10, *p* = 0.049). In older patients, the ORs and 95% CIs for the T2 and T3 groups compared with the T1 group were not significant (T2 vs. T1: OR 0.95, 95% CI 0.59–1.53, *p* = 0.830; T3 vs. T1: OR 1.43, 95% CI 0.88, 2.32, *p* = 0.153). The difference between the young and old groups was significant according to the interactive analysis (*p* for interaction = 0.013).

Supplementary Figure S1 describes a cubic spline curve illustrating the association between TGI levels and adjusted OR for 3 month death in all patients, young patients, and old patients.

Additionally, the logistic regression subgroup analysis is shown in Supplementary Table S1. Factors including stroke severity, sex, atrial fibrillation, rheumatic heart disease, coronary heart disease, diabetes, hypertension, cardioembolic subtype, lipid lowering therapy, antidiabetic drugs, and cancer had no effect on the association between TGI and 3 month death (all *p* for interaction>0.05).

The results also showed that TGI levels were not significantly associated with 3 month unfavorable functional outcomes in both younger and older groups (*p* > 0.05).

#### 3.2.2. TGI and 12 month outcomes

Similar results were found for 12 month death. An association was observed in younger patients (T3 vs. T1: OR = 2.52, 95% CI 1.21–5.22, *p* = 0.013). Conversely, in the older group, no association was presented between TGI and 12 month death (*p* > 0.05). According to the interactive analysis, the significant difference between the groups was observed (*p* for interaction = 0.027).

**Figure 1:**
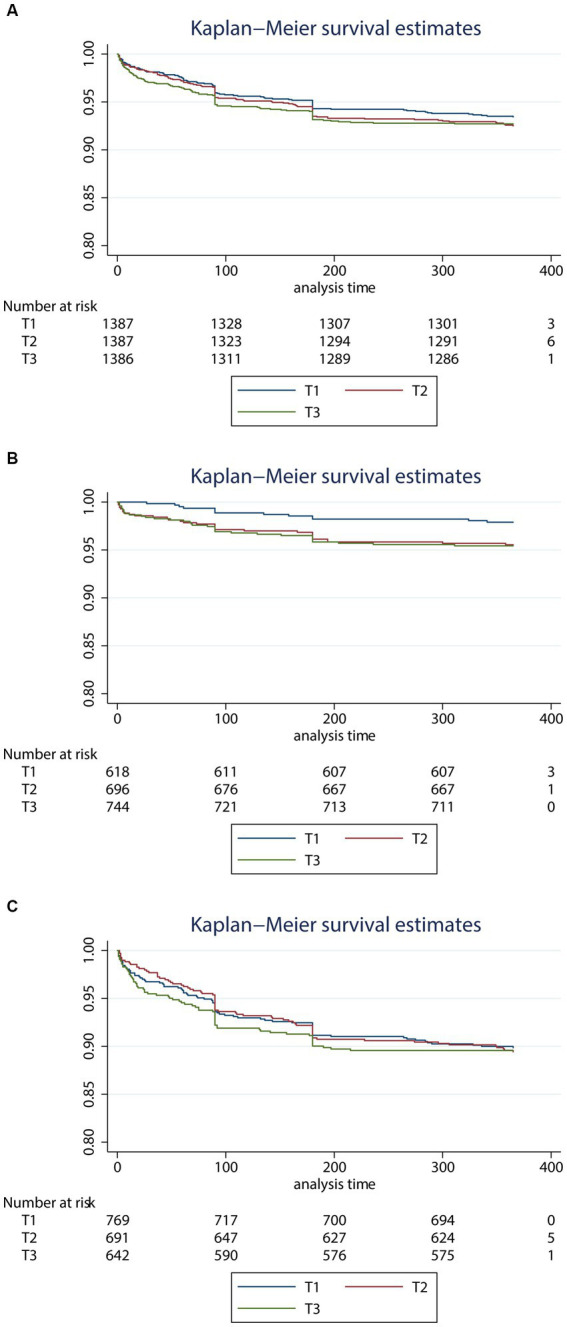
Kaplan–Meier survival estimates curve for the risk of 3 month death according to the level of triglyceride glucose index levels in all patients **(A)**, younger patients **(B)**, and older patients **(C)**. TGI, triglyceride-glucose index. **(A)** All patients, **(B)** younger patients, and **(C)** older patients.

These results were consistent with the Kaplan–Meier estimate analysis in all patients (Figure 1A), younger patients (Figure 1B), and older patients (Figure 1C). In the younger group, the 12 month death risk was higher in the group with high TGI (*p* for log-rank test = 0.028). While TGI was not significantly associated with 12 month death in the general people or the older people (*p* for the log-rank test = 0.691 and 0.979, respectively).

As shown in Supplementary Figure S2, a linear trend was observed for predicting 12 month death in the younger patients (*p* for trend = 0.012). For all patients and older patients, no significant linear association was observed (*p* for trend >0.05). In addition, no association was observed from interactive analysis between age and TGI on 12 month unfavorable functional outcome (*p* for interaction >0.05, Table 3).

## 4. Discussion

In this study, we explored the prognostic value of TGI levels among patients with ischemic stroke. We found that the association between TGI levels and the risk of death was mainly significant in patients younger than 65 years old. Younger patients with higher TGI levels exhibited an increased risk of death at 3 months and 12 months after stroke.

Although TGI might be associated with the prognosis of patients with stroke, the underlying mechanism is yet to be elucidated. Hyperglycemia in stroke patients predicts an unfavorable outcome, attributed to factors such as large infarct volume, hemorrhagic transformation, and recanalization failure (38–40). Hypertriglyceridemia contributes to early neurological deterioration and death primarily through atherosclerosis and cerebral vessel damage (41, 42). TGI, which may represent both hyperglycemia and hypertriglyceridemia, had better prognostic value than a single measurement alone. IR, as indicated by TGI, contributes to the development of more severe strokes and an increased risk of post-stroke death through various mechanisms. These mechanisms include proinflammatory cytokines induced by altered adiponectin signaling after stroke (43), and activation of hemostasis and a procoagulant tendency, leading to aggravated microvascular obstruction and cerebral ischemia (44).

Previous research has investigated the effect of TGI in selecting people at early risk of future stroke who are seemingly healthy (19–21). However, limited data are available regarding the association between TGI and death or functional outcomes in individuals with ischemic stroke, and the results are inconsistent (23–26). For example, high TGI was associated with a unfavorable functional outcome in Toh EMS’s study (24) but not in Zhou’s study (23). Yang et al. held a different opinion from others, reporting that TGI could not predict death in nondiabetic acute ischemic stroke patients (25). We propose that the discrepancy can be attributed, in part, to the interactive effect of TGI and other factors on stroke prognosis.

Studies have reported a greater susceptibility to IR among younger patients (45), and those with elevated TGI levels in this age group have a higher risk of IR-related diseases and death (27). Ma et al. discovered that patients below 70 years of age with elevated TGI levels exhibited an increased risk of cardiovascular and cerebrovascular diseases, including stroke, type 2 diabetes mellitus, and acute coronary syndrome, while no significant association was observed in older patients (46). Wang et al. suggested that a higher level of TGI in patients below 65 years of age with cardiovascular diseases may indicate an elevated risk of all-cause death (47). To explore the impact of age on the association between TGI and clinical outcome after stroke, we categorized the study population into older and younger groups. Interestingly, we observed a significant relationship between TGI and the risk of death at 3 months and 12 months in the younger group, while no such association was found in the older group. An interactive effect of age and TGI levels on the risk of death after stroke was observed. Additionally, a potential dose–response relationship between TGI and 12 month death was derived in younger patients based on our findings.

Therefore, we proposed that the role of IR in death may be significant in younger patients and that TGI was useful in predicting the prognosis in younger individuals with ischemic stroke. They may benefit from monitoring and treating IR.

There are several reasonable explanations for the effect of age on the association between TGI and the outcome. First, previous study suggested that younger individuals were more susceptible to IR (45), and had elevated TGI levels (45, 46, 48–50). On the contrary, an inverse association was observed in older patients: patients who were older had lower TGI levels, which was consistent with a previous study that explored the association between TGI and cardiovascular disease (48). For older individuals, TGI might not reflect IR the same as for younger individuals, probably because of more confounding factors that influence TGI measurement, such as prevalence of various chronic consumptive diseases and the subsequent malnutrition and decreased lipid reversal (51).

Moreover, based on the baseline information, higher TGI is associated with a lower proportion of heart disease, and associated with more large-artery atherosclerosis and less cardio-embolism subtype of stroke only in the younger group. Thus, for younger patients in our study, higher TGI reflected more severe atherosclerosis and worse vascular condition. A previous study has shown that for younger stroke patients, large-artery atherosclerotic subtype of stroke was a valuable predictor of worse outcomes including recurrence of vascular events, death, and unfavorable functional outcomes (52). Subgroup analysis based on etiologic stroke subtypes may help further explain our results.

Additionally, the predictive effect of TGI on functional outcome in individuals with stroke is still controversial. In our study, TGI might have had only a limited effect on unfavorable functional outcome in younger patients, which was different from the result regarding death. This was consistent with Zhou’s study (23) but opposite to Toh’s (24), and Lin’s (53) studies. Another study, which evaluated IR by HOMA-IR, confirmed that IR was associated with death, recurrence, and unfavorable functional outcome (an mRS score of 3 to 6) but not dependence (an mRS score of 3 to 5) in nondiabetic patients after stroke (54). Zhou interpreted that the different results concerning functional outcomes may result from the diversity of follow-up times, study design and race (23).

Possible explanations for the association between IR and death other than unfavorable outcomes are that IR is associated with the recurrence of ischemic stroke or cardiovascular disease in patients with stroke (25, 55), which is much more lethal than the first stroke. Nevertheless, some studies present opposite results, confirming that IR was associated with functional outcomes of poststroke patients (54, 56). However, they evaluated IR using HOMA-IR but not TGI, and the differences in IR marker measurements might account for the different findings. Additionally, stroke severity, the location of the lesion and diabetes condition also influenced functional outcomes (53, 57). Finally, the small sample size may also lead to controversial results. Further study based on a larger survey sample is needed to validate the results.

Significantly, TGI is a routinely measured and modifiable indicator to reflect the risk of post-stroke death compared with other markers. We propose suppression of IR and improvement of hyperglycemia or hypertriglyceridemia as new therapeutic strategies in patients with ischemic stroke to improve the prognosis. Since our study found that TGI has a greater impact on the outcome of younger patients, hyperglycemia and hypertriglyceridemia should be strictly controlled to prevent a higher risk of death, especially among young stroke patients. Pioglitazone, an insulin-sensitizing agent, as reported by several studies, is promising for reducing cardiovascular events in patients with ischemic stroke (58, 59). Other antidiabetic drugs, such as insulin, have been proven to inhibit the neuronal damage by suppressing elevated glucose levels within 48 h after ischemic stroke (60). Using these methods to regulate TGI, including taking drugs and modifying lifestyle, is expected to contribute to a better prognosis in post-stroke patients.

In this study, we explored the association between TGI and the outcomes of patients with ischemic stroke. This is the first study to analyze age as an interactive factor of the effect of TGI on the prognosis of ischemic stroke. The novelty and significance of this study were that we explored the possible interactive effect between age and TGI on the outcome of stroke. Besides, we explored the underlying reasons for the results that TGI affected post-stroke death but had few effects on post-stroke unfavorable functional outcomes for the first time. Although this was a retrospective study, inclusion bias and retrospective bias were adequately avoided with propensity-weighted analysis, continuous prospective follow-up, and a large sample size. Nevertheless, this study has several limitations. First, due to the low proportion of patients receiving reperfusion therapy (107 cases, 2.89%), including thrombolysis and endovascular treatment (MT), no further subgroup analysis by type of reperfusion therapy was performed. Previous studies have shown the association between TGI levels and the outcomes of patients undergoing thrombolysis (24, 53), while there were no studies on the effect of TGI on the outcomes in patients who underwent MT. According to Merlino’s study, stress hyperglycemia is strictly and independently associated with futile recanalization in patients undergoing MT (61). Thus, further study focus on the predictive value of TGI on the outcome of patients treated with MT is promising. Second, the median (Q1–Q3) time from stroke onset to blood sampling for TGI assessment is 88 (40–160) hours in the younger and 64 (40–136) hours in the older. Further subgroup analysis according to the time from onset to sampling is useful to detect the robustness of TGI as a serum marker. Fixing the time from onset to sampling or taking multiple blood samples from the same patient to observe the dynamic changes in serological indicators could make the results more convincing. Third, the retrospective nature of this study leads to inevitably residual or unmeasured confounding factors which may influence the prognosis, such as infarct size, etiological type, antithrombotic treatment at the admission and the baseline mRS socre. Besides, test for serum insulin, HOMA-IR, and hyperinsulinemic-euglycemic clamp help to measure IR in patients, which depends on further prospective studies.

## 5. Conclusion

Elevated TGI independently predicts death at 3 months and 12 months in patients under 65 with ischemic stroke. Therefore, TGI can be utilized as a supplementary test for prognosis assessment in young patients with ischemic stroke, while regulating TGI holds promise as an approach to enhance prognosis in young individuals affected by ischemic stroke.

## Data availability statement

The original contributions presented in the study are included in the article/Supplementary material, further inquiries can be directed to the corresponding authors.

## Ethics statement

The studies involving humans were approved by Biomedical Research Ethics Committee of West China Hospital, Sichuan University. The studies were conducted in accordance with the local legislation and institutional requirements. Written informed consent for participation was not required from the participants or the participants’ legal guardians/next of kin in accordance with the national legislation and institutional requirements.

## Author contributions

SZ and LW conceived and designed the research. RL and LL participated in analyzing the data and wrote the article. All authors contributed to the article and approved the submitted version.

## Funding

This study was supported by the National Natural Science Foundation of China (Grant nos. 81974208, 82202793, and 81501951) and Natural Science Foundation of Sichuan Province (Grant No. 2020YFC2008500, 2022NSFSC0715, and 2023NSFSC1495).

## Conflict of interest

The authors declare that the research was conducted in the absence of any commercial or financial relationships that could be construed as a potential conflict of interest.

## Publisher’s note

All claims expressed in this article are solely those of the authors and do not necessarily represent those of their affiliated organizations, or those of the publisher, the editors and the reviewers. Any product that may be evaluated in this article, or claim that may be made by its manufacturer, is not guaranteed or endorsed by the publisher.
