# Supplementary material for: Triglyceride-glucose index predicts death in patients with stroke younger than 65
Source: Front Neurol. 2023 Aug 3;14:1198487. doi: 10.3389/fneur.2023.1198487 (PMC10435085; doi:10.3389/fneur.2023.1198487)
Supplement: Supplementary file 1 [file Data_Sheet_1.pdf]

Supplementary Material

Triglyceride-Glucose Index Predicts 3-Month Death after Acute Ischemic Stroke in Patients of Different Ages

Ruishan Liu[1†], Lijuan Li[2,3†], Lu Wang[2,3*], Shihong Zhang[1*]

*** Correspondence:** Shihong Zhang: zsh9@hotmail.com; Lu Wang: wanglu0823@scu.edu.cn

Supplementary Table

Supplementary Table 1. Logistic regression subgroup analysis for the association between TGI as a categorical variable and 3-month death

**Complications**	**OR (95%CI), P-value**			**P for interaction**
	T1	T2	T3	
**Age**				**0.021**
Younger patients	1	2.65 (1.04, 6.72), 0.041	2.96 (1.16, 7.58), 0.024	
Older patients	1	0.94 (0.59, 1.50), 0.805	1.7 (0.79, 2.03), 0.323	
**Stroke severity**				0.715
Mild	1	1.00 (0.57, 1.76), 0.990	1.42 (0.82, 2.46), 0.211	
Severe	1	1.36 (0.74, 2.50), 0.317	1.45 (0.78, 2.73), 0.242	
**Sex**				0.099
Male	1	0.79 (0.43,1.45), 0.446	1.84(1.05, 3.22), 0.033	

Female	1	1.30(0.73, 2.34), 0.373	1.75(0.97, 3.15), 0.064	
**Atrial fibrillation**				0.783
Yes	1	1.26 (0.55,2.90), 0.584	2.10 (0.90,4.88), 0.084	
No	1	1.23(0.77, 1.98), 0.389	1.39(0.86, 2.25), 0.177	
**Rheumatic heart disease**				0.817
Yes	1	1.38(0.43,4.47), 0.590	1.21(0.29,5.12), 0.797	
No	1	1.14(0.74,1.76), 0.559	1.51(0.98,2.33), 0.060	
**Coronary heart disease**				0.899
Yes	1	2.62(0.55,12.44), 0.224	3.37(0.70,16.13), 0.129	
No	1	1.16(0.76,1.78), 0.487	1.40(0.91,2.16), 0.131	
**Diabetes**				0.559
Yes	1	1.21(0.40,3.62), 0.733	0.97(0.33,2.81), 0.954	
No	1	1.08(0.69,1.69), 0.731	1.55(0.96,2.50), 0.071	
**Hypertension**				0.610
Yes	1	1.17(0.68,2.02), 0.573	1.36(0.79,2.33), 0.272	

No	1	1.17(0.63,2.18), 0.624	1.78(0.92,3.45), 0.089	
**Cardioembolic stroke**				0.384
Yes	1	1.36(0.14,13.21), 0.791	4.67(0.51,42.57), 0.171	
No	1	1.11(0.74,1.68), 0.616	1.35(0.87,2.09), 0.181	
**Lipid lowering therapy**				0.317
Yes	1	0.94(0.56,1.58), 0.818	1.47(0.90,2.39), 0.126	
No	1	1.65(0.82,3.34), 0.159	1.74(0.81,3.75), 0.154	
**Antidiabetic drugs**				0.593
Yes	1	1.13(0.46,2.76), 0.796	1.09(0.46, 2.62), 0.839	
No	1	1.14(0.72,1.81), 0.570	1.73(1.07, 2.80), 0.025	
**Cancer**				0.387
Yes	1	14.35 (0.61, 339.06） 0.099	151.79(1.93, 11949.1） **0.024**	
No	1	1.19 (0.78, 1.80) 0.425	1.24 (0.90, 1.91) 0.344	

Adjusted for age, sex, baseline NIHSS, the time from stroke onset to admission, atrial fibrillation, rheumatic heart disease, coronary heart disease, drinking, smoking, TOAST, hemoglobin, creatinine, HDL, antiplatelet therapy.

Supplementary Figures

Supplementary Figure 1. Cubic spline curve for the association between TGI levels and adjusted odds ratio for 3-month death in all patients (Supplementary Figure 1A), young patients (Supplementary Figure 1B), and old patients (Supplementary Figure 1C).

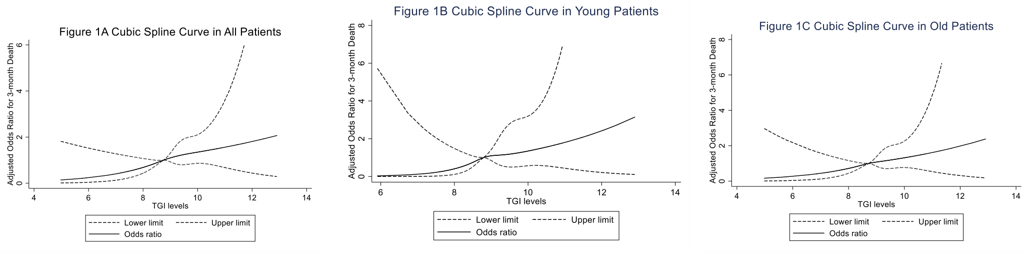

Logistic regression analyses with adjustment for age, sex, baseline NIHSS, the time from stroke onset to admission, atrial fibrillation, rheumatic heart disease, coronary heart disease, drinking, smoking, TOAST, hemoglobin, creatinine, HDL, antiplatelet therapy.

Supplementary Figure 2. Cubic spline curve for the association between TGI levels and adjusted odds ratio for 12-month death in all patients (Supplementary Figure 2A), young patients (Supplementary Figure 2B), and old patients (Supplementary Figure 2C).

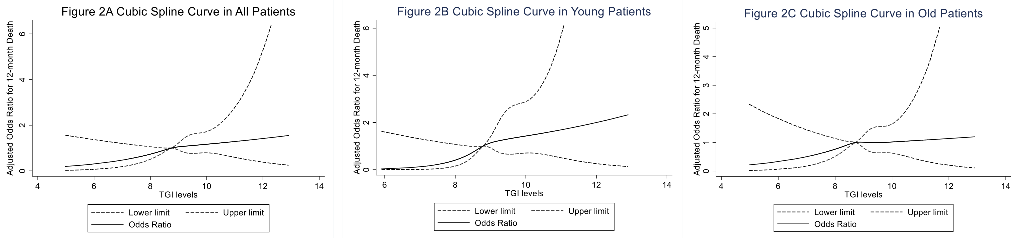

Logistic regression analyses with adjustment for age, sex, baseline NIHSS, the time from stroke onset to admission, atrial fibrillation, rheumatic heart disease, coronary heart disease, drinking, smoking, TOAST, hemoglobin, creatinine, HDL, antiplatelet therapy.
